# Electrolysis in reduced gravitational environments: current research perspectives and future applications

**DOI:** 10.1038/s41526-022-00239-y

**Published:** 2022-12-05

**Authors:** Ömer Akay, Aleksandr Bashkatov, Emerson Coy, Kerstin Eckert, Kristian Etienne Einarsrud, Andreas Friedrich, Benjamin Kimmel, Stefan Loos, Gerd Mutschke, Lars Röntzsch, Mark D. Symes, Xuegeng Yang, Katharina Brinkert

**Affiliations:** 1 Center for Applied Space Technology and Microgravity (ZARM), University of Bremen, 28359 Bremen, Germany; 2 Department of Physics, Free University Berlin, 14195 Berlin, Germany; 3 Technische Universität Dresden, Institute of Process Engineering and Environmental Technology, D-01062 Dresden, Germany; 4 Helmholtz-Zentrum Dresden-Rossendorf, Institute of Fluid Dynamics, Bautzner Landstraße 400, D-01328 Dresden, Germany; 5 NanoBioMedical Centre, Adam Mickiewicz University, Wszechnicy Piastowskiej 3, 61-614 Poznan, Poland; 6 Department of Materials Science and Engineering, NTNU Norwegian University of Science and Technology, 7034 Trondheim, Norway; 7 Institute of Engineering Thermodynamics, German Aerospace Center, Pfaffenwaldring 38-40, Stuttgart, 70569 Germany; 8 Fraunhofer Institute for Manufacturing Technology and Advanced Materials IFAM, Branch Lab Dresden, Winterbergstraße 28, 01277 Dresden, Germany; 9 Brandenburg University of Technology BTU, Hydrogen Research Center, 03046 Cottbus, Germany; 10 WestCHEM, School of Chemistry, University of Glasgow, Glasgow, G12 8QQ UK; 11 Department of Chemistry, University of Warwick, Coventry, CV4 7AL UK

**Keywords:** Electrocatalysis, Electrochemistry, Chemical physics

## Abstract

Electrochemical energy conversion technologies play a crucial role in space missions, for example, in the *Environmental Control and Life Support System* (ECLSS) on the *International Space Station* (ISS). They are also vitally important for future long-term space travel for oxygen, fuel and chemical production, where a re-supply of resources from Earth is not possible. Here, we provide an overview of currently existing electrolytic energy conversion technologies for space applications such as proton exchange membrane (PEM) and alkaline electrolyzer systems. We discuss the governing interfacial processes in these devices influenced by reduced gravitation and provide an outlook on future applications of electrolysis systems in, e.g., in-situ resource utilization (ISRU) technologies. A perspective of computational modelling to predict the impact of the reduced gravitational environment on governing electrochemical processes is also discussed and experimental suggestions to better understand efficiency-impacting processes such as gas bubble formation and detachment in reduced gravitational environments are outlined.

## Introduction

Sustaining life on Earth and in space is dependent on the existence of suitable energy sources and chemical building blocks such as molecular oxygen, water and carbon sources. Water electrolysis represents one possibility for converting electrical energy into chemical energy by producing oxygen and hydrogen. It has therefore been of key interest to the space research community as human space exploration relies on an efficient and stable supply of oxygen. The first investigations of water electrolyzers for space applications date back to the 1960s^1,2^, when the main obstacles with developing the technology for space applications were identified: on Earth, the gravitational acceleration gives rise to buoyancy which leads to the detachment of gas bubbles from the electrode surface and a separation of oxygen and hydrogen gas bubbles from the liquid electrolyte. Given the near-absence of buoyant forces in reduced gravitational environments, alternative strategies (e.g., rotating water electrolyzers) have been investigated to artificially introduce gravitation and phase-separation^3,4^, although this is associated with an additional energy cost. Water electrolysis has been investigated intensively in microgravity environments (10^−2 ^*g*–10^−6 ^*g*) over the past three decades in order to increase the efficiency of devices utilized on spacecrafts and on the *International Space Station* (ISS) and to understand the governing interfacial processes at the electrode-electrolyte interface^5–8^. Here, we review the state-of-the-art developments of (photo-)electrolyzer systems for space applications, elucidate the key processes influenced by the environment and suggest experiments and computational models to improve the understanding and efficiency of these devices.

## Application of electrolyzer systems in space

**Fig. 1:**
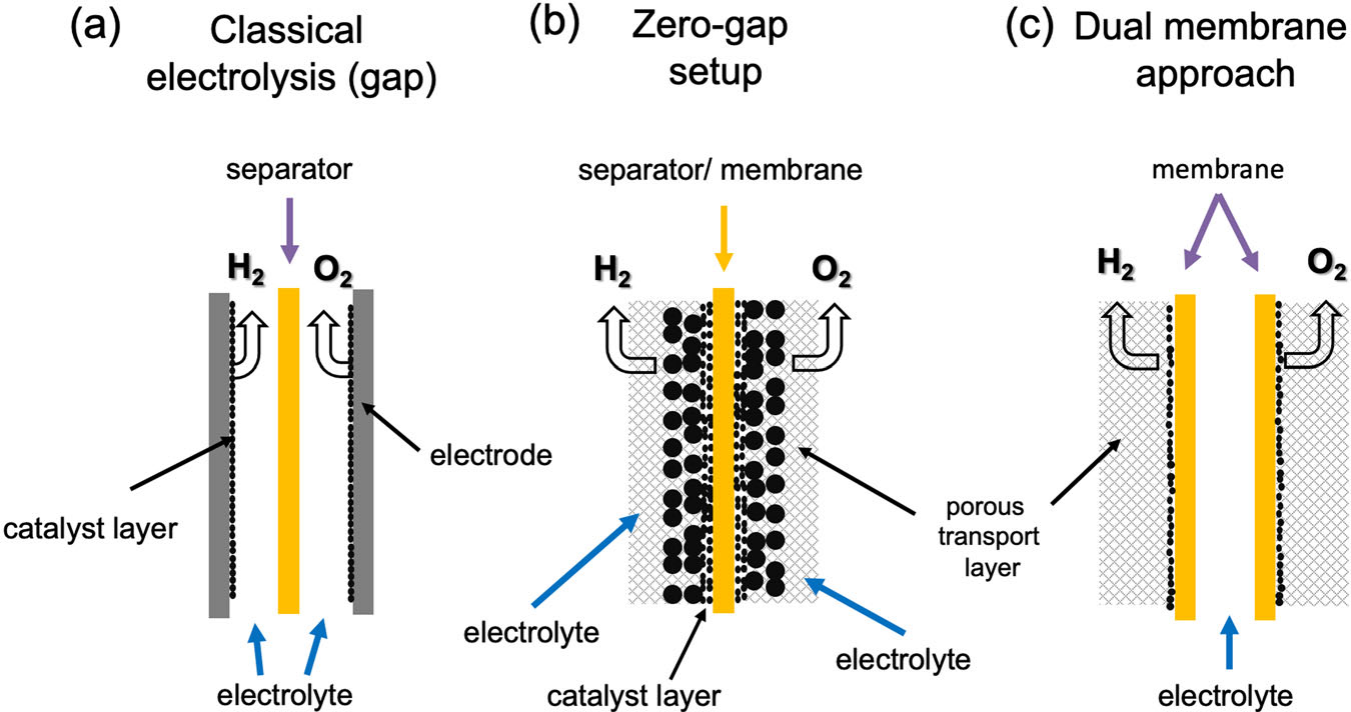
Design of electrolyzer cells. **a** Classical electrolysis setup with a separator and two electrodes separated by a finite distance, (**b**) zero-gap setup with catalysts and porous transport layers and (**c**) dual-membrane cell which could potentially be suitable for low-g applications. In contrast to layouts (**a**) and (**b**), liquid electrolyte/water is fed in layout (**c**) between two membrane sheets.

From a practical point of view, gas and water management in water electrolyzer cells is crucial for both, terrestrial and space applications, and a high purity of reactants and products is required. Both criteria are governed by the cell design. Figure 1a shows a classical electrolysis cell with gaps between electrodes and separator (e.g., a membrane), whereas a zero-gap arrangement (Fig. 1b) does not include a gap between electrode and separator. The latter set-up typically results in larger current densities and higher cell efficiencies as liquid electrolyte resistance does not add to the ohmic losses. It requires however porous electrodes and transport layer structures and gas crossover is an increasingly relevant issue^9^. The structures of the porous transport layer (PTL) and the catalytic layer in conjunction with the electrolyte flow need to mediate the gas formation and the bubble detachment. In order to facilitate efficient gas bubble detachment, the electrode and the separator/membrane are required to stay wet and possess a high degree of hydrophilicity as gases in moist environments have the tendency to accumulate on hydrophobic surfaces. Although this set-up would be very interesting for space applications given the high achievable efficiencies, it is particularly challenging to realize as these requirements are difficult to address in space environments. An attempt has been made where two membranes are used instead of one (Fig. 1c). Here, the electrolyte flows in between them and gases can be released to the separate chambers on the right and left of the membranes away from the liquid^10,11^. In this way, the cathode and anode chambers can be kept free of any liquid electrolyte and the gases can be removed easily. In order to achieve high efficiencies and low energy footprints in the dual-membrane approach, the electrochemical accessible surface area of the electrode has to be increased, which is usually achieved by utilizing a PTL and instead of planar electrodes, foams, fibres, meshes or felts. These materials offer the porosity required for a zero-gap operation and can enhance the accessible surface by a factor of roughly 3–50. In addition, the implementation of high surface area catalyst coatings with enhanced intrinsic activities such as nanoparticles, Raney-type structures and microstructures is of interest as they add to the surface area and lead to relative roughness factors up to a few thousand^12,13^.

Although these points also hold for microgravity environment, the cell design, feeds and flow rates, electrodes structures, separator characteristics as well as PTL structures require adaptation and need to be revisited completely for this purpose due to the absence of buoyancy.

### Proton exchange membrane electrolyzer

**Fig. 2:**
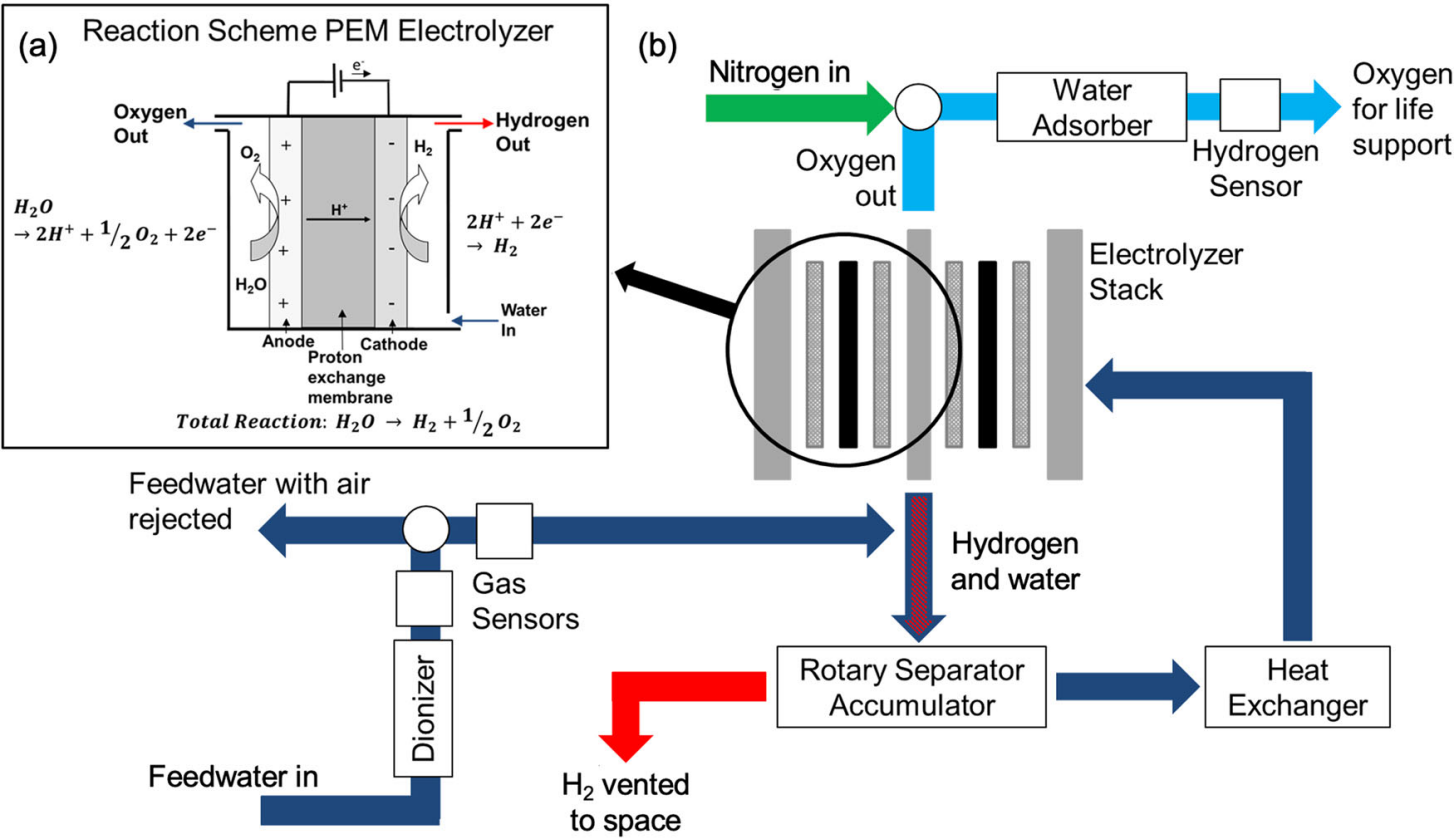
Oxygen production on the International Space Station (ISS). **a** Design of a PEM electrolyzer cell (cathode-feed, inset) and (**b**) the integration into the Oxygen Generator Assembly (OGA) on the ISS.

The most prominent application of electrolyzer systems in space lies in the provision of oxygen in the *Environmental Control and Life Support System* (ECLSS). Oxygen is produced by a low temperature proton exchange membrane (PEM) electrolyzer within the *Oxygen Generation Assembly* (OGA), Fig. 2^14^. Very little is publicly known about the precise design and operating conditions of the PEM electrolyzer on the ISS, but publications of initial, terrestrial experiments allow a rough characterization. A 13-cell stack is utilized with an active area of 214 cm^2^ per cell following a layout similar to Fig. 1b. Cathode water feed operation with a flow rate of 8.07 kg/hr and an inlet temperature of 339 K leads to an average stack temperature of 335 K^15^. The average pressure is 2860 kN m^−2^ with a small overpressure on the anode side. The stack itself is operated at 75 A, corresponding to 350 mA cm^−2^ with an average cell voltage of 1.72 V^15^. Pt based catalysts designated as E-50 by *General Electric*^8,16^ are used on the anode side and Pt Black is utilized on the cathode side^15^. The PEM electrolyzer can produce between 2.3 and 9.2 kg of oxygen per day at a selectable rate in continuous operation. In terms of energy consumption, improvements to the OGA are of high interest due to its high power demand relative to the entire ECLSS (around 33%)^14^.

Carbon dioxide in cabin air is removed and converted by a Sabatier reactor which produces methane and water. While methane is not used on the ISS at this time, the produced water is extremely valuable and can be reused in the OGA^17^.

In order to reduce the necessary amount of payload water, the company Astrium recently developed a closed-loop air revitalization system called *ARES* and successfully demonstrated its functionality under terrestrial conditions. The main components of the system are an electrolyzer, a Sabatier reactor and a CO_2_ removal assembly^17,18^. In ARES, an alkaline electrolyzer with an immobile (non-flowed) electrolyte is used and liquid water and product gases are directly separated^18,19^. However, PEM electrolyzers possess advantages over alkaline electrolyzers which explains their implementation in the current OGA system^14^: they exhibit higher current and power densities and operate at lower temperatures, which results in higher overall efficiencies and lower energy footprints. They also produce gases with a higher purity over a large operating window^14^.

### Fray-Farthing-Chen (FFC)—Cambridge process

**Fig. 3:**
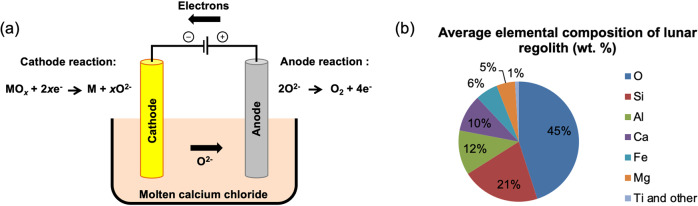
The FFC-Cambridge process and its potential application on the moon. **a** The principle behind the FFC-Cambridge process for the electrochemical reduction of metal oxides to give the metals and oxygen. **b** An approximate average elemental composition (wt. %) of lunar regolith serving as the cathode material.

The Fray-Farthing-Chen (FFC)—Cambridge process constitutes an alternative electrochemical method to the electrolysis of H_2_O for obtaining oxygen on extra-terrestrial bodies such as the moon and Mars. This process was originally developed for the extraction of metals such as titanium and tantalum from their oxides^20^, and allows the direct reduction of solid metal oxides to their corresponding metals (also in the solid state), without the need to melt the oxide. Instead, a molten salt electrolyte (usually calcium chloride) at temperatures of around 900 °C is used as a selective and effective conductor of O^2−^ ions from the cathode (where the metal oxide is reduced) to the anode (see Fig. 3a). Certain anode materials (e.g., tin oxides) are then able to discharge these O^2−^ ions to give oxygen gas.

A small number of studies have shown that such a process is a viable method of generating oxygen electrochemically from artificial simulants of lunar soils (“lunar regolith”), co-generating reduced (metallic) regolith species as by-products (which could find use in construction on the lunar surface)^21–23^. Moreover, the FFC-Cambridge process could offer a number of unique benefits over alternative methods for oxygen production in certain contexts. For example, on the moon, a layer of regolith 5–10 m thick covers the entire surface, and (as shown in Fig. 3b), oxides of silicon, aluminium and calcium comprise the majority of this material^24^. Lunar regolith is therefore a promising feedstock for oxygen production via the FFC-Cambridge process on the moon (whereas water-ice seems to exist only at the poles)^25^.

### Electrolytic CO_2_ reduction

Continuous oxygen supply and efficient air recycling are critical aspects for long-term space exploration and missions. The most widely explored and implemented solutions for the reduction of carbon dioxide on board spacecraft are the Sabatier and Bosch reactions. Both processes catalytically reduce gaseous CO_2_ with H_2_ and enable the recovery of metabolic oxygen. These reactions have been actively researched and implemented since the early 1950s and their implementation as viable oxygen sources took the rest of the 20th century, with a fully integrated reactor completed only in 2003^26^. In the Sabatier reaction, CO_2_ reacts with hydrogen to give methane and water^27,28^, whilst the Bosch reaction produces water and elemental carbon^29^:

1 $${{{\mathrm{CO}}}}_2\left( {{{\mathrm{g}}}} \right) + {{{\mathrm{4H}}}}_{{{\mathrm{2}}}}\left( {{{\mathrm{g}}}} \right) \leftrightarrow {{{\mathrm{CH}}}}_{{{\mathrm{4}}}}\left( {{{\mathrm{g}}}} \right) + {{{\mathrm{2H}}}}_{{{\mathrm{2}}}}{{{\mathrm{O}}}}\left( {{{\mathrm{l}}}} \right)\;\;\left( {{{{\mathrm{Sabatier}}}}} \right)$$

2 $${{{\mathrm{CO}}}}_{{{\mathrm{2}}}}\left( {{{\mathrm{g}}}} \right) + {{{\mathrm{H}}}}_{{{\mathrm{2}}}}\left( {{{\mathrm{g}}}} \right) \to {{{\mathrm{CO}}}}\left( {{{\mathrm{g}}}} \right) + {{{\mathrm{H}}}}_{{{\mathrm{2}}}}{{{\mathrm{O}}}}\left( {{{\mathrm{l}}}} \right)\;\;\left( {{{{\mathrm{Bosch}}}}\;{{{\mathrm{Step}}}}\;{{{\mathrm{1}}}}} \right).$$

3 $${{{\mathrm{CO}}}}\left( {{{\mathrm{g}}}} \right) + {{{\mathrm{H}}}}_{{{\mathrm{2}}}}\left( {{{\mathrm{g}}}} \right) \to {{{\mathrm{C}}}}\left( {{{\mathrm{s}}}} \right) + {{{\mathrm{H}}}}_{{{\mathrm{2}}}}{{{\mathrm{O}}}}\left( {{{\mathrm{l}}}} \right)\;\;\left( {{{{\mathrm{Bosch}}}}\;{{{\mathrm{Step}}}}\;{{{\mathrm{2}}}}} \right).$$

Although the Sabatier reactor vents the produced CH_4_ with some loss of O_2_ and H_2_, it is currently in use on the ISS as the Bosch process shows slower reaction rates, the fouling of catalyst materials due to carbon deposits and less favorable thermodynamics due to the endothermic reverse water gas shift reaction in Step 1. The Sabatier reactor operates at about 515 °C and employs an alumina-supported ruthenium catalyst which has a higher selectivity toward methane than the traditionally used nickel catalyst^26^.

A new, emerging field of research is the electrolytic reduction of CO_2_ in solid oxide electrolysis cells (SOEC) to directly produce oxygen as part of in-situ *resource utilization* (ISRU) technologies developed for Mars exploration^30,31^. The Martian atmosphere contains about 96% CO_2_^32^, making it a prime resource for oxygen extraction. SOECs are attractive because of unrivaled conversion efficiencies which are a result of favorable thermodynamics and kinetics at high operating temperatures (600–850 °C). A dense, ceramic electrolyte such as yttria (Y_2_O_3_)—stabilized zirconia (ZrO_2_, YSZ) capable of conducting oxide ions (O^2−^) is used together with a Ni-YSZ cathode^31^. For less demanding reactions, oxygen electrodes based on Earth abundant Sr-doped LaMnO_3_ (LSM) can be used, whereas higher performing applications require electrodes based on mixed conductors such as lanthanum-strontium-ferrite-cobaltite (LSCF)^31^. The anode is usually covered in a thin layer of gadolinia-doped ceria (CGO) to prevent the reaction of oxygen with the electrode materials and YSZ. The Mars 2020 Perseverance Rover included a SOEC as part of a so-called “Mars Oxygen In-Situ Resource Utilization Experiment” (MOXIE). MOXIE could produce O_2_ at a rate of 10 g/h and a purity level of 99.6% [https://mars.nasa.gov/mars2020/spacecraft/instruments/moxie/ (accessed 28/04/2022)], utilizing a doped-lanthanum cobalt ferrite anode and a Ni-ceria cermet cathode for CO_2_ reduction:

4 $${{{\mathrm{Cathode}}}}:{{{\mathrm{2CO}}}}_{{{\mathrm{2}}}} + {{{\mathrm{4e}}}}^ - \to {{{\mathrm{2CO}}}} + {{{\mathrm{2O}}}}^{{{{\mathrm{2 - }}}}}$$

5 $${{{\mathrm{Anode}}}}:{{{\mathrm{2O}}}}^{{{{\mathrm{2 - }}}}} \to {{{\mathrm{O}}}}_{{{\mathrm{2}}}}+{{{\mathrm{4e}}}}^{{{\mathrm{ - }}}}$$

6 $${{{\mathrm{Overall}}}}\;{{{\mathrm{cell}}}}\;{{{\mathrm{reaction}}}}:{{{\mathrm{2CO}}}}_{{{\mathrm{2}}}} \to {{{\mathrm{2CO}}}} + {{{\mathrm{O}}}}_{{{\mathrm{2}}}}$$

Side reactions include however the production of elemental carbon at the cathode which deposits on the subjacent Ni catalyst. SOECs are also investigated for water electrolysis. Due to the high operating temperatures, gaseous water is fed into the cells which avoids obstacles related to gas bubble evolution.

### Photoelectrocatalytic oxygen and chemical production

Photoelectrochemical devices take inspiration from natural photosynthesis and are currently being developed for terrestrial carbon dioxide reduction and simultaneous water oxidation^33^. They employ integrated semiconductor-electrocatalyst systems combining light absorption, charge separation and transfer as well as electrocatalysis^34^. Due to their monolithic design, they are interesting for space applications as they offer significant weight and volume advantages in comparison to traditional photovoltaic-powered electrolyzer systems currently employed on the ISS. The high system tunability allows the synthesis of a large variety of products at the anode and cathode. Whereas water oxidation can be chosen as a primary reaction at the (photo-)anode for oxygen generation, the photocathode could be utilized for carbon dioxide reduction to relevant hydrocarbon-based propellants or the production of nitrogen-based fertilizers from the reduction of atmospheric dinitrogen. Another advantage is the ease with which the electrocatalyst-liquid interface can be engineered e.g., for efficient gas bubble detachment in reduced gravitation, as discussed in the next section. Current developments of photoelectrochemical devices for terrestrial applications aim at operation in aqueous electrolytes at near-neutral pH which also represents a safety advantage^34^. Photoelectrolytic half-cells for hydrogen production have been tested in drop tower experiments where they could operate at terrestrial efficiencies^35,36^.

## Electrochemical gas bubble evolution

In space environments, electrolysis systems are severely influenced by hindered detachment of gas bubbles from the electrode due to the near-absence of buoyancy. This is known to induce overpotentials^37^ due to increased ohmic resistances in proximity to the electrode caused by gas bubbles in the electrolyte and the blockage of bubble nucleation sites, as well as a reduced product and reactant mass transfer. As a result, the overall efficiency of the electrochemical system decreases. Removing gas bubbles from the electrode surface rapidly, efficiently and at small sizes is a major challenge in the near-absence of buoyant forces.

### Gas bubble nucleation and growth

When considering a single bubble, its evolution proceeds via a four-step process, nucleation, growth, coalescence and finally detachment^38,39^. The nucleation phase starts when the local supersaturation of dissolved gas sufficiently exceeds equilibrium concentration^40,41^. Despite the multifaceted nature of the nucleus formation, the nucleation phase is less affected by reduced gravity than the growth and detachment phases. The growth of a single bubble is governed by diffusion^42–44^ and by coalescence with smaller bubbles^45–48^ which form a micro-layer at the electrode^49^. The increase of the bubble radius *R* with time typically follows a power law *R*(*t*) ~ *t*^*x*^^48,50^. Depending on whether diffusion or coalescence is the dominant growth mechanism, the power exponent *x* varies between 1/2 and 1/3, and may even approach 1/4 at microelectrodes.

### Electrode wettability and bubble departure

The wettability of electrodes is already a crucial aspect to consider even in terrestrial catalysis and photocatalysis as it directly influences the efficiency of the processes and impacts the turnover frequency of reactions^51^. The wettability can determine if, on one hand, an active center is blocked for further reactions by strongly adsorbing bubbles or, on the other hand, if the surface has repelled the gas bubbles by attracting the fluids and is ready for further catalytic processes. Wettability reaches its utmost priority in low gravitational environments, where buoyancy does not contribute to the bubbles’ release. The wettability of liquids is inversely related to the wettability of bubbles since hydrophilic surfaces allow rapid wetting by liquids and keep bubbles at relatively high contact angles, preventing them from blocking active catalytic centers^52^. The relationship follows the equation described by Young (1805)^53^:

7 $$\sigma _{{\it{SG}}} = \sigma _{SL} + \sigma _{LG}{{{\mathrm{cos}}}}\;\theta$$

**Fig. 4:**
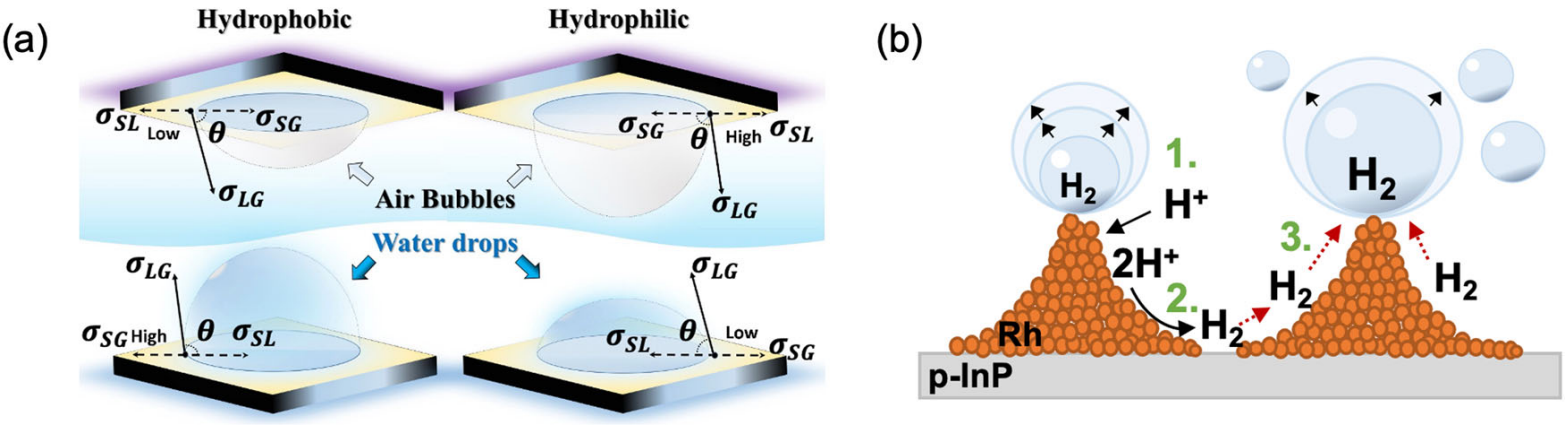
Electrode surface modifications for increased gas bubble detachment in microgravity. **a** Scheme illustrating the difference between hydrophobic and hydrophilic surfaces when in contact with (air) gas bubbles (top) and water droplets (bottom). **b** Diagram of the improved mechanism for hydrogen bubble nucleation and reduced bubble coalescence on nanostructured photoelectrode surfaces in a microgravity environment according to ref. ^35^.

Here, *σ*_*SL*_, *σ*_*SG*_ and *σ*_*LG*_ refer to the specific surface energy of the solid-liquid and the solid-gas interfaces, respectively, as well as to the surface (liquid-gas) tension of the bubble; *θ* is the contact angle. Notice the inverted relation between bubbles and droplets, Fig. 4a. Equation (7) defines the microscopic contact angle, which can be influenced by modifying the surface tension coefficients, *σ*_*ij*_, e.g., by changing materials or locally changing the material properties, see below. Besides, as already mentioned above, the wettability can be further influenced by tailoring the surface shape of the electrode, see e.g., Fig. 4b. The wettability of plane and modified surfaces with respect to hydrogen and oxygen bubbles in microgravity conditions was studied by Akay et al. (2021), Brinkert et al. (2018) and Sakuma et al. (2008, 2014)^35,36,46,54^.

**Fig. 5:**
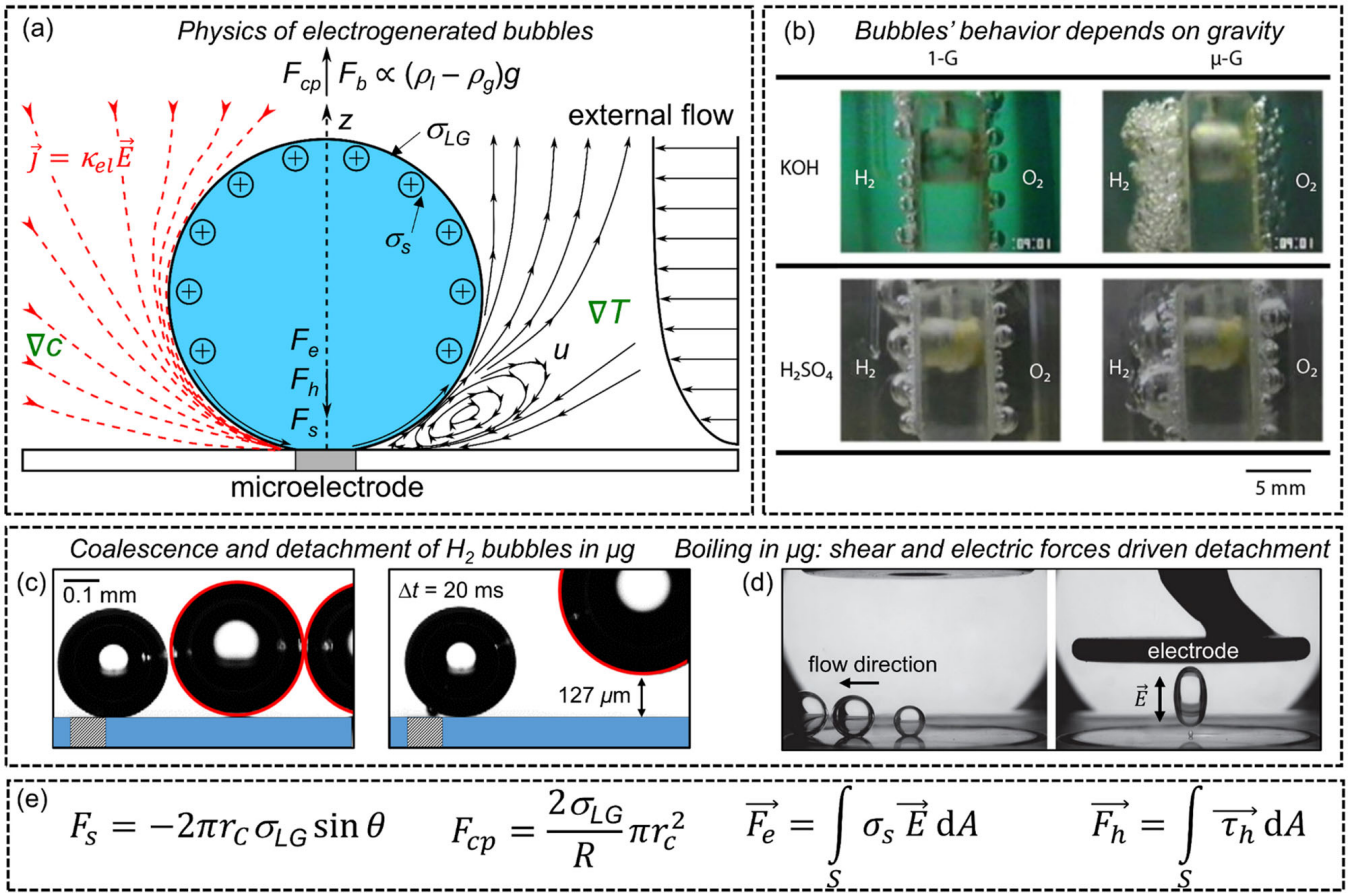
Gas bubble evolution in terrestrial and reduced gravitational environments. **a** Scheme representing the physics of electrogenerated bubbles on microelectrodes. Red lines are the electric current lines directed from anode to cathode. The black lines show schematically the Marangoni convection at the bubble foot as observed in Yang et al. (2018)^68^. If the electrolyte’s pH value is below the isoelectric point, the surface charge density of the bubble, *σ*_*S*_, is positive as shown in (**a**), and negative otherwise^114^. **b** Evolution of oxygen and hydrogen gas bubbles in terrestrial (left column) and in micro-g (right column) in alkaline (−0.8 V vs. RHE, 25 wt.% KOH) and acidic (−0.8 V vs. RHE, 0.1 N H_2_SO_4_) electrolytes. Reprinted from Electrochim. Acta, 48/28, Matsushima et al. Water electrolysis under microgravity: Part 1. Experimental technique, 4119–4125, 2003, with permission from Elsevier^57^. **c** Two hydrogen bubbles (circled in red) in microgravity shortly before their coalescence (left). The energy released by coalescence forces the resultant bubble to jump by ca. 130 µm normal to the electrode surface (right) from Bashkatov et al. (2021)^63^. **d** Comparison of bubble behavior during boiling with (**a**) shear flow and (**b**) shear flow with electric field according to ref. ^67^. **e** Collection of the different forces acting on the bubble in (**a**).

The gas bubble experiences several forces (see Fig. 5a) which vary in magnitude during growth. The bubble eventually detaches when the liberating forces, most importantly buoyancy, overcome the adhesive forces between bubble and electrode, namely the electrostatic force *F*_*e*_, the hydrodynamic force *F*_*h*_ and the surface tension force *F*_*s*_^55^. As shown in Fig. 5e, they mainly depend on the bubble’s surface charge density *σ*_*s*_, the electric field *E*, the stress tensor *τ*_*h*_ integrated over the bubble’s surface *A* and on the surface tension *σ*_*LG*_. The detachment of the bubble sets in via disconnecting the bubble neck from the electrode^37^ or by losing contact with the carpet of microbubbles underneath^49^.

The dynamics of hydrogen and oxygen bubbles in microgravity were studied on macro-sized electrodes at drop towers^35,36,54,56–60^, during parabolic flights^61–63^, on a sounding rocket^5^ and on the ISS^6^. As buoyancy is considerably reduced in microgravity, gas bubbles remain longer on the electrode and grow to larger size, thus forming a gas layer on macro-sized electrodes^6,57,58,61^, see Fig. 5b. This decreases the effective area of the electrocatalyst, impedes mass transport and increases the reaction overpotential^36^. To avoid the obvious complexity arising from the interaction of multiple bubbles, several studies on the dynamics of single hydrogen or oxygen bubble were performed on microelectrodes^46,63,64^.

The detachment of individual bubbles from the electrode is typically triggered by coalescence events, where the liberated energy causes sudden bubble jumps away from the surface^6,56–58,63,65^ as depicted in Fig. 5c. Coalescence events in microgravity may also cause lateral reversals of bubble motion, thus encouraging lateral detachment from microelectrodes^63^. The importance of bubble-bubble interaction for bubble detachment on a micro-pin-finned electrode was also discussed by Zhou et al. (2018) in subcooled pool boiling experiments^66^.

The detachment mechanism can be further facilitated by surface functionalization toward hydrophilicity^35,36^, for example as reported on nanostructured photoelectrodes (see below), or by using shear and electrostatic forces as studied on board of the ISS during boiling processes^67^, see Fig. 5d. This effect is further discussed below. Generally, pressurized electrolyzers also lead to smaller gas bubbles which are easier to remove from the electrodes’ surfaces.

At low current densities, the adsorption of dissolved hydrogen is faster on hydrophobic than on hydrophilic surfaces^36^. Hence, when the bubble growth is diffusion-controlled, e.g., in the absence of buoyancy, they are expected to grow faster. Furthermore, soluto- or thermocapillary forces^68–70^ caused by gradients of species, ∇*c*, as well as temperature, ∇*T*, see Fig. 5a and Lorentz forces^71–73^, originating from a combination of electric and magnetic fields, might be reasonable instruments to enhance the detachment of the gas bubbles.

Porous electrodes are known for their large electrochemical active surface areas and their applicability in (zero-gap) electrochemical reactors. They provide a higher number of nucleation sites compared to planar electrodes and show faster gas bubble detachment due to smaller contact angles^74^. A challenge for gas bubble removal might arise however from gas bubbles trapped inside the pores of the electrocatalysts and the PTLs in microgravity.

### Computational modelling perspectives on gas bubble growth and detachment in microgravity

The description of electrochemical gas bubble evolution depends on a multiscale and multiphysics approach^75^, spanning several orders of magnitude in both space and time^38,76^. The accurate prediction of nucleation requires knowledge of the distribution of active nucleation sites on the electrode surface as well as that of dissolved species in the electrolyte. Such phenomena are well beyond the continuum scale, and rely upon atomistic scale modelling, i.e., molecular dynamics^77,78^. While atomistic and molecular scale models in principle could handle the growth step, the computational cost of such an approach quickly becomes prohibitive when considering more than one bubble or length scales larger than a few µm. The different scales can be bridged by adopting fluid parcel-based methods such as the *Lattice Boltzmann Method*^79,80^ and *Smoothed Particle Hydrodynamics*^81^, in which interactions between fluid parcels, or particles, which consist of several molecules are considered. Recently, approaches based on *Computational Fluid Mechanics* (CFD), which traditionally have been used to study coalescence and detachment, have successfully been demonstrated for growth and evolution of 100 µm bubbles^82^, indicating that the borders between the various simulation strategies are gradually becoming less strict as increased computational capacity becomes available.

The accurate description of bubble detachment relies upon determining the balance between adhesive forces between bubble and electrode, i.e., interfacial tension, and forces promoting bubble escape as discussed above^38^, i.e., buoyancy, growth forces and drag forces, but also forces owing to electrostatics and Marangoni convection^70^ as schematically shown in Fig. 5a, e. Under terrestrial conditions, the detachment is typically determined by interfacial tension and buoyancy^39^, leading to models for bubble detachment radius derived on this basis^83^. Accurate prediction of bubble departure remains however challenging, indicating that the models applied so far require further improvement by considering all relevant forces. Models which describe, for instance, the detachment diameter are critical input for large scale CFD models, typically based on Eulerian-Lagrangian or Eulerian-Eulerian approaches^84^, aiming to simulate electrochemical processes on a system level^85^. Evidently, the development of such closure laws under low- and microgravity conditions is critical for future simulation-based optimization.

In practice, electrochemical devices evolve multiple bubbles on the electrodes^60,86,87^, which interact in a complex, nonlinear manner, reaching a so-called ‘self-organized state’. Self-organization depends strongly on convection^88^, which evidently depends on buoyancy or lack thereof. This is inherently linked to convection-induced drag forces which challenge adhesion to the electrodes, but also to convective heat and mass transfer, which ultimately governs the growth and nucleation of future bubbles. Numerical simulations of such systems rely on a multiscale approach in which various simulation strategies are coupled to bridge the scales. For instance, population balance modelling can be coupled to interface resolving methods, as demonstrated for aluminium electrolysis^89^. In the extreme case of zero gravity, secondary, e.g., Marangoni^70^, and even tertiary sources of convection such as electrostatic contributions^38^ or contributions from the electrical double layer^90^ can dominate. These phenomena can contribute significantly also in terrestrial systems as long as buoyancy is small, i.e., in the initial growth stage. Generally, they are however challenging to quantify. As such, experiences gained in low- and zero-gravity environments will further advance our understanding of the initial conditions for bubble evolution also on Earth.

## Efficiency enhancements of electrochemical reactions in microgravity

### Nanopatterning solutions

**Fig. 6:**
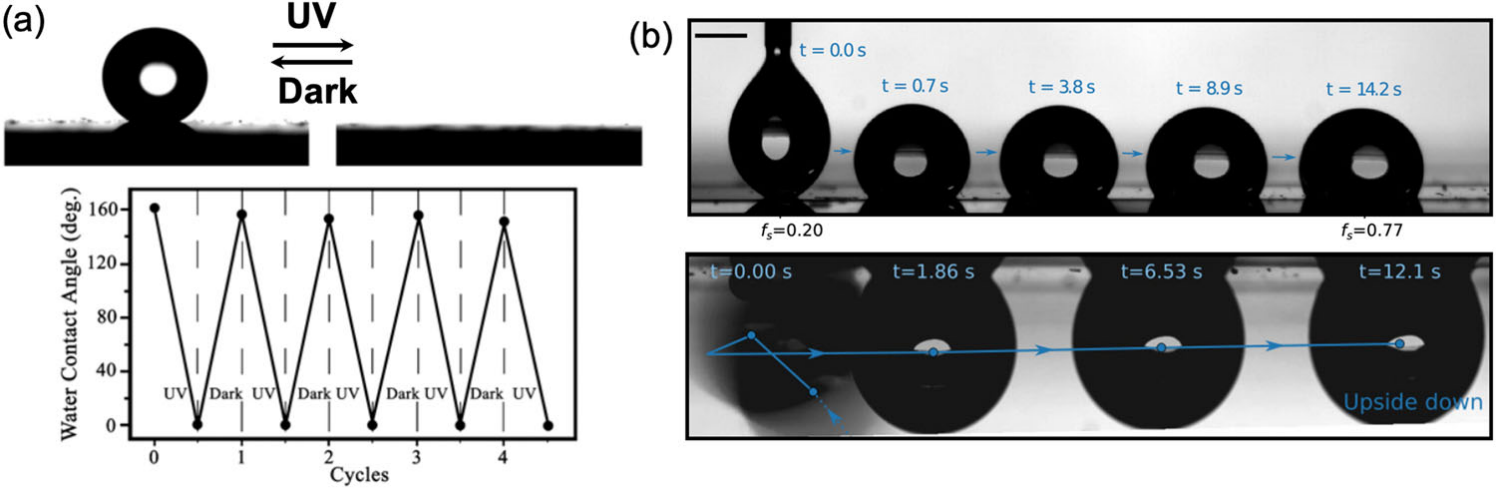
Strategies for enhanced gas bubble detachment. **a** Illustration demonstrating the variations of the water contact angle during UV irradiation cycles. Reprinted with permission from Feng, X. et al. Reversible super-hydrophobicity to super-hydrophilicity transition of aligned ZnO nanorod films. J. Am. Chem. Soc. **126** (1), 62–63 (2004). Copyright 2004, American Chemical Society^96^. **b** Illustration of a time lapse imagery of a self-propelled water droplet on a nanopatterned surface (top). A similar illustration from a study of a droplet suspended upside down on a patterned surface (bottom) from Launay et al. (2020)^98^.

Among several possible solutions for the bubble detachment issue in reduced gravitational environments, nanopatterning of surfaces is one of the most promising. Several advancements in the nanopatterning of surfaces have been explored in the literature, resulting in super hydrophilic/-phobic surfaces^91,92^. However, many of these strategies are difficult to extrapolate to active electrode materials as they involve functionalization and passivation of surfaces. One of the most widely used approaches for increasing the wettability of photoelectrodes is the activation of surfaces by UV irradiation (i.e., on TiO_2_, ZnO, etc.)^93^ as shown in Fig. 6a. This approach has been shown to work well and it is especially attractive in microgravity environments where unfiltered solar radiation is plentiful. A critical study on different TiO_2_ surfaces showed changes in contact angle from ~32.5° down to ~2.5°, and almost 0° during longer exposure^94^. Similar studies on ZnO films confirmed its similar behavior, although, the study also showed the reversibility of the process in the absence of UV-radiation^95^ as shown in Fig. 6a^96^. This poses a challenge and raises questions of the efficiency of this approach in *in-operando* conditions, which has not been studied in microgravity environments. Moreover, the effect of the continuous generation of superoxides, reactive oxygen species, and the electrodes’ photocorrosion must also be addressed. Nanopatterning of surfaces has been tested in microgravity experiments using nanostructured thin-film electrodes. The general approach involves the selective deposition of catalytic centers through a sacrificial lithography mask, typically on a micrometer scale. This methodology resulted in a more significant separation of nucleation centers for bubble formation, and (possibly more critical for space applications) the dramatic reduction of the bubble coalescence rate as shown in Fig. 6b^35^.

The possibility of tailoring and applying surfaces that allow for the self-propelled movement of droplets and bubbles might play an important role in future technologies^97^. Several advancements in microfluidic and lab-on-chip technologies have explored the possibility of wettability-assisted propulsion. Experimental evidence shows the transport of droplets over surfaces held at an angle and even upside down, see Fig. 6b^98^. Testing the capabilities of this approach for both, liquids and bubbles in microgravity conditions, might help address froth growth, coalescence, and the blockage of the electrodes’ active centers^74^. The preferable design approach for an efficient (photo-)electrochemical system in a microgravity environment is therefore the consideration of surface-functionalized electrodes, further assisted by shear, magnetic, electric, and Lorentz forces as well as thermocapillary induced convection as introduced in the Section *Electrochemical Gas Bubble Evolution*.

### Phase separation for electrochemical devices

When operating electrolyzers in microgravity environments, a key challenge is the separation of the gas and liquid phases in the absence of buoyancy^15^. In electrolyzers, the liquid water is normally injected into the anode side where oxidation occurs. Therefore, gaseous oxygen exists in liquid water. Gravity-based separators are used terrestrially, but become inefficient under microgravity conditions. Gravity-independent separation is much more complex, and several approaches have been developed in the past^99,100^. One way to achieve low-gravity phase separation is to use centrifugal pumps in combination with cooling pumps and pipes as currently implemented on the ISS^14,15^. Another, more advantageous system to improve reliability and lifespan is passive phase separation using porous glass membrane separators. Further efforts are however necessary to improve passive phase separation in general e.g., with respect to the required membrane size^15,16^. Alongside improved separation processes, an alternative approach is to inject water only on the cathode side to avoid mixing gaseous oxygen and liquid water^8,15,16^. Gaseous hydrogen and liquid water still need to be separated to prevent water loss, especially when the hydrogen is vented (as it is currently the case on the ISS)^14,17^. Beside these approaches, in which the gaseous phase is separated from liquid water, there are several attempts in which water vapor is utilized instead of liquid water. Water vapor can be fed into the system carried by gaseous argon^15,19^. The insertion of argon however also results in difficulties in obtaining pure hydrogen. Generally, the utilization of water vapor also leads to the disadvantageous effect of parasitic power consumption which is required to generate the vapor^15^. An approach to avoid these problems is a static water feed system using porous membranes^8,15^. Liquid water is driven in this system by concentration gradients and diffuses across the porous membrane. It reaches the active side of the electrolyzers in form of water vapor. The disadvantage of this system is a rather low rate of oxygen production, which is limited by the water diffusion rate^15^. Although, the combination of static water feed and phase separation is more advantageous than a direct water feed with active phase separation, only the active method has been applied in the ECLSS on the ISS^15^. Further investigations on different approaches to separate the gas-water phase are still of great interest and importance for manned space missions.

Another important research topic is the optimization of the gas/water management within the PEM electrolyzer in microgravity as gas bubbles hardly detach from the electrode. Furthermore, a pumping effect caused by the buoyant rise of gas bubbles is not expected^8^. Terrestrially, PTLs are used to ensure a good gas/water management and are therefore an essential part of electrolyzers to enable high performance as discussed above^98,99^. The PTLs have to provide good electrical conductivity while transporting species toward and away from the catalytic layer^98^. At high current densities with higher gas production rates, the property of PTLs to keep mass transport losses low is becoming increasingly important for the performance of electrolyzers^99^. On the anode side, titanium is mainly used as the base material due to its stability in the acidic environment in PEM electrolyzers^8,19^. On the cathode side, carbon-based structures like carbon papers are commonly used as PTLs^8,19^. For application in microgravity environments, new designs are however required to ensure a high electrolyzer performance and long lifespan.

## Outlook: Open research questions, experiment suggestions and future technologies

Electrolysis will very likely play a major role in human space exploration as it offers the unique possibility of converting abundant sunlight into vital chemicals for humans. This section explores open questions in the research field with respect to the application of (photo-)electrolyzer systems in space, suggests experiments in reduced gravitational and hypergravity environments which could enhance the understanding of the relevant processes involved and discusses current, technological developments which could be relevant for future space applications^101^. Generally, the successful development of efficient electrolysis systems for non-terrestrial applications will certainly also go hand-in-hand with pushing their terrestrial utilization, where high efficiencies and low costs are also of key importance.

### Electrocatalytic reduction of gaseous reactants

Gaseous reactants like CO_2_ and N_2_ represent a particular challenge for electrolytic devices in microgravity environment as the separation of gases and liquids is hindered as discussed above. In microgravity environments and at high gas flow rates, gas bubbles in liquids follow a so-called ‘slug-annual’ flow pattern, where large, continuous gas bubbles with a smooth interface and a spherically shaped nose flow through the liquid separated by liquid slugs^102^. With an increasing gas velocity, the liquid slug decreases in length up to a collapse, so liquid flows as a film at the wall of the reaction vessel or tube and the gas flows in the center^102^. Therefore, the gas flow rate has to be carefully adjusted in reduced gravitational environments to ensure that the electrolyte is still in contact with the electrode surface during the electrocatalytic reduction of (e.g.,) CO_2_. Microgravity represents however also an opportunity to investigate the electrocatalytic reduction of gaseous reactants in more detail, given the low solubility of CO_2_ and N_2_ in aqueous solution under terrestrial conditions [https://www.engineeringtoolbox.com/gases-solubility-water-d_1148.html (accessed 29/04/2022)]. The near-absence of buoyancy could result in the reactants accumulating close to the electrode surface. Initial tests regarding the gas interaction with the electrode surface could be carried out in drop tower and parabolic flight experiments.

### Low *g* and molten salts

To date, experimental studies on the bubble-forming reactions in the FFC-Cambridge process are lacking. This leaves a number of open questions as to how bubble formation in the FFC-Cambridge process is influenced by the electrode material, especially as the use of inert, oxygen-generating electrodes for the FFC-Cambridge process is in its infancy when compared to the more established use of carbon electrodes (which generates CO and CO_2_, and so is less attractive for space applications where oxygen would be a much more valuable product). Furthermore, there appear to be no experimental studies to date on the effects of low gravity on any molten salt electrolysis process. These constitute some key knowledge gaps which will need to be filled before, say, an electrolyzer using FFC-Cambridge technology could be deployed on the lunar surface.

The viscosity of molten calcium chloride at 900 °C (as employed in the FFC-Cambridge process) is ~2.3 mPa s^103^, roughly the same as that of whole milk at 20 °C (~2.1 mPa s) and not entirely dissimilar to that of water at 0 °C (in the region of 1.8 mPa s). Given the considerable technical challenges associated with conducting any detailed experimental study of bubble behavior in molten salts at high temperatures, let alone interrogating such systems under reduced gravity, it may be that studying aqueous solutions at near-ambient temperature could serve as a useful and more accessible proxy for FFC-Cambridge electrolysis at high temperatures. With this in mind, Lomax et al. (2022) recently investigated the influence of gravity on the oxygen evolution reaction during electrochemical water-splitting at ambient temperature across the gravitational range 10^−2^ – 8 *g* using electrolysis cells mounted on a centrifuge^86^. By operating the system during parabolic flights, *g* levels below 1 could be obtained. Alternatively, employing an identical system in ground-based experiments allowed *g* levels above 1 to be achieved. By careful comparison of the trends obtained under both, hypergravity and during parabolic flight, the authors were able to show that data obtained using ground-based centrifuges (delivering 1–8 *g*) could be extrapolated to Martian and lunar gravity. This finding has the potential to significantly simplify studies into electrolysis systems that might be deployed on the moon or Mars, by allowing ground-based systems (generally much less expensive and more accessible than parabolic flights) to be used to predict the characteristics of an electrolysis system operating under reduced gravity. This in turn may help to make the study of molten salt electrolysis under reduced gravity somewhat more feasible, as flying a molten salt electrolyzer on a parabolic flight is likely to be fraught with technical and safety regulatory challenges.

### Reversible alkaline electrolyzer/fuel cell and other future water

#### Electrolyzer technologies

Combining an electrolyzer with a fuel cell in one device could enable the operation in a reversible fashion — a regenerative or reversible electrochemical cell. Very little is known about such a technology and it became rather silent recently, but a proof of concept has been published^104–106^. Relying on non-platinum group metals only (e.g., Ni, Mn, Fe, Cu, Co) will however be difficult to achieve. In particular, the catalyst selection for the reversible mode is challenging as the electrochemical window for operation spans oxygen evolution and reduction as well as the hydrogen evolution reaction. Furthermore, electrodes in a reversible cell will have to cope with liquid electrolyte in the electrolysis and with (moist) gases in the fuel cell mode at the same time. The device could be powered by solar energy when water electrolysis is performed to produce vital molecular oxygen. It could also power heating and cooling systems as well as electrical light when hydrogen is being oxidized in order to produce clean water. Such a system would be unique and would require a complete conceptual chemical and technological understanding. The development of such a system for a non-terrestrial application would certainly also accelerate terrestrial developments.

In recent years, a few very innovative approaches have appeared in the electrolysis literature which might be especially useful for microgravity environments. The concepts are more or less far away from the classical idea of electrolysis and involve for example (1) separation of hydrogen and oxygen evolution in space and time using redox mediators^107^, (2) electron-coupled proton buffers^108,109^, (3) dissolution and electrodeposition processes^110^ and (4) the combination of electrochemical and chemical steps for water alkaline electrolysis^111^. These variants can sometimes achieve very low crossover, high efficiencies and they can operate without a separator unit.

A second branch of innovative electrolysis concepts is related to ‘bubble-free’ operation in a capillary-fed electrolysis cell^112^, which avoids ohmic losses by bubbles as gas is extracted before bubbles are formed. These systems typically exhibit low onset potentials and have very high efficiencies.

### Gas-water separation

To overcome the need for gas-water separation on the oxygen side, cathode water feed is a very attractive alternative since fewer system parts are required. According to the current state-of-the-art, however, only low current densities can be achieved with this method. A limiting factor is the diffusion rate of water between the cathode and anode sides. The capability of the membrane to absorb water is also crucial for good conductivity and heat transfer. Therefore, further material studies are required to achieve higher current densities to increase the oxygen production rate without the need to upscale the systems. A prototype of a water electrolysis system for terrestrial research with the possibility of using different directions of gravity will be beneficial in obtaining first estimations of performances before its evaluation in microgravity environments.

### Porous transport layers

Higher current densities increase the importance of the removal of gaseous products from within electrolysis cells^113^. For operation under microgravity conditions, optimized PTLs are required for enhanced gas bubble detachment. New designs, materials and optimized pore structures can however only be tested and verified in microgravity. The analysis of different PTLs can be supported using special cell setups with windows to evaluate the capability of the PTLs to remove bubbles.

This review outlines some of the key challenges and open research questions associated with the application of (photo-)electrolyzers in reduced gravitational environments. Given the key importance of these systems for human space exploration, it should also provide an inspiration and encouragement to the broader research community to investigate these systems in micro- and hypergravity environments further and develop approaches to solutions.

## Data Availability

All relevant data are available from the authors.

## References

[1] Clifford, J. E., McCallum, J., Gates, J. T. & Faust, C. L. *Research on the electrolysis of water under weightless conditions*. Tech. Rep. AMRL-TDR-62-44 (NASA, 1962).

[2] Newman, D. Water electrolysis reaction control system. In: *Proc. of the 7th Liquid Propulsion Symposium*, 105–114 (Chemical Propulsion Information Agency Publications 72, 1965).

[3] Erickson, R. J., Howe J., Kulp, G. W. & Van Keuren, S. P. International space station united states orbital segment oxygen generation system on-orbit operational experience. In: *International Conference On Environmental Systems*, 2008-01-1962 (2008).

[4] Samplatsky, D. J. & Dean, W. C. Development of a rotary separator accumulator for use on the International Space Station, in: *International Conference On Environmental Systems* (SAE International, 2002).

[5] Wopersnow W, Raub JC (1978). Elektrolytische Wasserstoffentwicklung under Schwerelosigkeit. Z. Flugwiss. Weltraumforsch..

[6] Nefedov VG (1994). Gas evolution and behaviour of gas phase in water electrolysis under conditions of weightlessness. Russ. J. Electrochem..

[7] Subramanian, R.S. & Balasubramaniam, R. *The motion of bubbles and drops in reduced gravity* (Cambridge University Press, 2001).

[8] Sakurai M, Sone Y, Nishida T, Matsushima H, Fukunaka Y (2013). Fundamental study of water electrolysis for life support in space. Electrochim. Acta.

[9] Kienzlen V, Haaf D, Schnurnberger W (1994). Location of hydrogen gas evolution on perforated plate electrodes in zero gap cells. Int. J. Hydrog. Energy.

[10] Walter, J., Lucas, J. & Raatschen, W. Method for operating an electrolysis cell. EP2765224 (A1), patent issued August 13^th^, 2014. https://patents.google.com/patent/DE4120679A1/en (Accessed 25 Nov 2022).

[11] Raatschen, W., Lucas, J., Jehle, W. & Funke, H. Electrolysis method and electrolysis cells. EP2463407 (A1), patent issued June 13^th^, 2012. https://patents.google.com/patent/EP2463407A1/en (Accessed 25 Nov 2022).

[12] Fujita T (2015). Nanoporous metal papers for scalable hierarchical electrode. Adv. Sci..

[13] Bernäcker CI, Rauscher T, Büttner T, Kieback B, Röntzsch L (2019). A Powder metallurgy route to produce raney-nickel electrodes for alkaline water electrolysis. J. Electrochem. Soc..

[14] Raymond, C., Nelson, G. J. & Perry, J. L. Electrolyzer exergy analysis for an environmental control and life support system, IMECE2018-88119, V06AT08A068.

[15] Qing G, Fang Y, Guo H, Ma CF (2017). Gas/water and heat management of PEM-based fuel cell and electrolyzer systems for space applications. Microgravity Sci. Technol.

[16] Sakurai, M., Shima, A., Sone, Y. & Ohnishi, M. Development of oxygen generation demonstration on JEM (KIBO) for manned space exploration. 44th ICES Tuscon, Arizona, USA,13–17 July 2014. American Institute of Aernoautics and Astronautics 10.2514/6.2013-3449.

[17] Raatschen, W. Ventilation and air revitalization on the International Space Station (ISS). 22^nd^ Annualy AIVIC-Conference Bath, United Kingdom, 11–14 September 2001. https://www.aivc.org/sites/default/files/members_area/medias/pdf/Conf/2001/Raatschen%20Ventilation%20on%20the%20ISS.pdf (Accessed 25 Nov 2022).

[18] Feustel-Büechl, J. The Newsletter of the Directorate of Human Spaceflight and Microgravity - ESA **14**, September 2003. https://www.esa.int/esapub/onstation/os14.pdf (Accessed 25 Nov 2022).

[19] Spurgeon JM, Lewis NS (2011). Proton exchange membrane electrolysis sustained by water vapor. Energy Environ. Sci..

[20] Chen GZ, Fray DJ, Farthing TW (2000). Direct electrochemical reduction of titanium dioxide to titanium in molten calcium chloride. Nature.

[21] Schwandt C, Hamilton JA, Fray DJ, Crawford IA (2012). The production of oxygen and metal from lunar regolith. Planet. Space Sci..

[22] Lomax BA (2020). Proving the viability of an electrochemical process for the simultaneous extraction of oxygen and production of metal alloys from lunar regolith. Planet. Space Sci..

[23] Meurisse A (2022). Lower temperature electrochemical reduction of lunar regolith simulants in molten salts. Planet. Space Sci..

[24] McKay, D. S. et al. The lunar regolith, (eds Heiken, G.H., Vaniman, D.T. & French, B.M.), *Lunar Sourcebook* 285–356 (Cambridge University Press, 1991).

[25] Li S (2018). Direct evidence of surface exposed water ice in the lunar polar regions. Proc. Natl Acad. Sci..

[26] Greenwood, Z. W., Abney, M., B., Brown, B. R., Fox, E. T. & Stanley, C. State of NASA Oxygen Recovery. 48th ICES Albuquerque, New Mexico, USA, 8-12 July NASA (on behalf of the International Conference on Environmental Systems, 2018).

[27] Vogt C, Monai M, Kramer GJ, Weckhuysen BM (2019). The renaissance of the Sabatier reaction and its applications on Earth and in space. Nat. Catal..

[28] Murdoch, K., Goldblatt, L., Carrasquillo, R. & Harris, D. Sabatier methanation reactor for space exploration. In: *1st Space Exploration Conference: Continuing the Voyage of Discovery* (American Institute of Aeronautics and Astronautics, 2005).

[29] Green, R. D. et al. Development Status for a Combined Solid Oxide Co-Electrolyzer and Carbon Formation Reactor System for Oxygen Regeneration. In: *AIAA SPACE 2016* (American Institute of Aeronautics and Astronautics, 2016).

[30] Gunduz S, Deka DJ, Ozkan US (2018). Chapter three - advances in high-temperature electrocatalytic reduction of CO_2_ and H_2_O. Adv. Catal..

[31] Hecht M (2021). Mars oxygen ISRU experiment (MOXIE). Space Sci. Rev.

[32] Hartvigsen JJ (2015). Challenges of solid oxide electrolysis for production of fuel and oxygen from Mars atmospheric CO_2_. ECS Trans..

[33] Zhou X (2016). Solar-driven reduction of 1 atm of CO_2_ to formate at 10% energy-conversion efficiency by use of a TiO_2_-protected III-V tandem photoanode in conjunction with a bipolar membrane and a Pd/C cathode. ACS Energy Lett..

[34] Cheng W-H (2018). Monolithic photoelectrochemical device for 19% direct water splitting. ACS Energy Lett.

[35] Brinkert K (2018). Efficient solar hydrogen production in microgravity environment. Nat. Commun..

[36] Akay Ö (2021). Releasing the bubbles: nanotopographical electrocatalyst design for efficient photoelectrochemical hydrogen production in microgravity environment. Adv. Sci.

[37] Angulo A (2020). Influence of bubbles on the energy conversion efficiency of electrochemical reactors. Joule.

[38] Taqieddin A, Allshouse MR, Alshawabkeh AN (2018). Editors’ Choice-Critical Review-Mathematical Formulations of Electrochemically Gas-Evolving Systems. J. Electrochem. Soc..

[39] Zhao X, Ren H, Luo L (2019). Gas Bubbles in Electrochemical Gas Evolution Reactions. Langmuir.

[40] German SR (2018). Critical nuclei size, rate, and activation energy of H_2_ gas nucleation. J. Am. Chem. Soc..

[41] Battistel A (2019). Local study on hydrogen and hydrogen gas bubble formation on a platinum electrode. J. Phys. Chem..

[42] Westerheide DE, Westwater JW (1961). Isothermal growth of hydrogen bubbles during electrolysis. AIChE J..

[43] Brandon NP, Kelsall GH (1985). Growth kinetics of bubbles electrogenerated at microelectrodes. J. Appl. Electrochem..

[44] Soto ÁM, Lohse D, van der Meer D (2020). Diffusive growth of successive bubbles in confinement. J. Fluid Mech..

[45] Verhaart HFA, De Jonge RM, Van Stralen SJD (1980). Growth rate of a gas bubble during electrolysis in supersaturated liquid. Int. J. Heat. Mass Trans..

[46] Sakuma G, Fukunaka Y, Matsushima H (2014). Nucleation and growth of electrolytic gas bubbles under microgravity. Int. J. Hydrog. Energy.

[47] Fernandez D (2014). Bubble formation at a gas-evolving microelectrode. Langmuir.

[48] Yang X (2015). Dynamics of single hydrogen bubbles at a platinum microelectrode. Langmuir.

[49] Bashkatov A (2019). Oscillating hydrogen bubbles at pt microelectrodes. Phys. Rev. Lett..

[50] Brandon NP, Kelsall GH (1985). Growth kinetics of bubbles electrogenerated at microelectrodes. J. Appl. Electrochem..

[51] Ardagh MA, Abdelrahman OA, Dauenhauer PJ (2019). Principles of Dynamic Heterogeneous Catalysis: Surface Resonance and Turnover Frequency Response. ACS Catal..

[52] Li T (2019). The Effect of Surface Wettability and Coalescence Dynamics in Catalytic Performance and Catalyst Preparation: A Review. Chem. Cat. Chem..

[53] Young T (1805). An essay on the cohesion of fluids. Philos. Trans. R. Soc..

[54] Sakuma G, Matsushima H, Fukunaka Y (2008). Interfacial phenomena of bubble evolution in water electrolysis under microgravity. Int. J. Microgravity Sci. Appl..

[55] Thorncroft GE, Klausner JF, Mei R (2001). Bubble forces and detachment models. Multiph. Sci. Technol..

[56] Iwasaki A (1998). Investigation of electrochemical hydrogen evolution under microgravity condition. Electrochim. Acta.

[57] Matsushima H (2003). Water electrolysis under microgravity: Part 1. Experimental technique. Electrochim. Acta.

[58] Matsushima H, Fukunaka Y, Kuribayashi K (2006). Water electrolysis under microgravity: Part II. Description of gas bubble evolution phenomena. Electrochim. Acta.

[59] Kiuchi D (2006). Ohmic resistance measurement of bubble froth layer in water electrolysis under microgravity. J. Electrochem. Soc..

[60] Sides P. J. Phenomena and effects of electrolytic gas evolution. *Modern Aspects of Electrochemistry*. 303–354 (Springer, Boston, MA, 1986).

[61] Kaneko H (1993). Water electrolysis under microgravity condition by parabolic flight. Electrochim. Acta.

[62] Mandin P (2014). Bubble over-potential during two-phase alkaline water electrolysis. Electrochim. Acta.

[63] Bashkatov A (2021). Dynamics of single hydrogen bubbles at Pt microelectrodes in microgravity. Phys. Chem. Chem. Phys..

[64] Matsushima H (2009). Single bubble growth during water electrolysis under microgravity. Electrochem. Commun..

[65] Soto ÁM (2018). Coalescence of diffusively growing gas bubbles. J. Fluid Mech..

[66] Zhou J, Zhang Y, Wei J (2018). A modified bubble dynamics model for predicting bubble departure diameter on micro-pin-finned surfaces under microgravity. Appl. Therm. Eng..

[67] Sielaff A (2022). The multiscale boiling investigation on-board the International Space Station: An overview. Appl. Therm. Eng..

[68] Yang X, Baczyzmalski D, Cierpka C, Mutschke G, Eckert K (2018). Marangoni convection at electrogenerated hydrogen bubbles. Phys. Chem. Chem. Phys..

[69] Massing J (2019). Thermocapillary during hydrogen evolution at microelectrodes. Electrochim. Acta.

[70] Hossain SS, Mutschke G, Bashkatov A, Eckert K (2020). The thermocapillary effect on gas bubbles growing on electrodes of different sizes. Electrochim. Acta.

[71] Baczyzmalski G (2016). On the electrolyte convection around a hydrogen bubble evolving at a microelectrode under the influence of a magnetic field. J. Electrochem. Soc..

[72] Mutschke G (2017). Numerical simulation of mass transfer and convection near a hydrogen bubble during water electrolysis in a magnetic field. Magnetohydrodynamics.

[73] Baczyzmalski D (2017). Growth and detachment of single hydrogen bubbles in a magnetohydrodynamic shear flow. Phys. Rev. Fluids.

[74] Li C (2021). Dynamic self-propelling condensed microdroplets over super-hydrophobic surface: an exceptional atmospheric corrosion inhibition strategy. Colloids Surf. A Physicochem. Eng. Asp..

[75] Weinan, E. *Principles of Multiscale Modeling*. (Cambridge University Press, 2011).

[76] Taqieddin A, Nazari R, Rajic L, Alshawabkeh A (2017). Review-Physicochemical hydrodynamics of gas bubbles in two phase electrochemical systems. J. Electrochem. Soc..

[77] Maruyama S, Kimura T (1999). A Molecular Dynamics Simulation of a Bubble Nucleation on Solid Surface. Trans. Jpn. Soc. Mech. Eng. B.

[78] Hofbauer F, Frank I (2012). Electrolysis of Water in the Diffusion Layer: First-Principles Molecular Dynamics Simulation. Chem. Eur. J..

[79] Nemati M, Shateri Najaf Abady AR, Toghraie D, Karimipour A (2018). Numerical investigation of the pseudopotential lattice Boltzmann modeling of liquid-vapor for multi-phase flows,”. Phys. Stat. Mech. Its Appl..

[80] Gong S, Cheng P (2013). Lattice Boltzmann simulation of periodic bubble nucleation, growth and departure from a heated surface in pool boiling. Int. J. Heat. Mass Transf..

[81] Nugent S, Posch HA (2000). Liquid drops and surface tension with smoothed particle applied mechanics. Phys. Rev. E.

[82] Vachaparambil KJ, Einarsrud KE (2021). Numerical simulation of continuum scale electrochemical hydrogen bubble evolution,”. Appl. Math. Model..

[83] Oguz HN, Prosperetti A (1993). Dynamics of bubble growth and detachment from a needle. J. Fluid Mech..

[84] Jakobsen, H. A. Multiphase Flow. In: *Chemical Reactor Modeling: Multiphase Reactive Flows*, Jakobsen, H.A. Ed. 369–536 (Cham:Springer International Publishing, 2014).

[85] Le Bideau D (2020). Eulerian Two-Fluid Model of Alkaline Water Electrolysis for Hydrogen Production. Energies.

[86] Lomax BA (2022). Predicting the efficiency of oxygen-evolving electrolysis on the Moon and Mars. Nat. Commun.

[87] Sequeira CAC, Santos DMF, Šljukić B, Amaral L (2013). Physics of Electrolytic Gas Evolution. Braz. J. Phys..

[88] Orlik, M. Convection as a Source of Self-Organization in Electrochemical Systems. In: *Self-Organization in Electrochemical Systems II: Spatiotemporal Patterns and Control of Chaos*, (ed Orlik M.) 265–374 (Springer, Berlin, Heidelberg, 2012).

[89] Einarsrud KE (2017). Towards a coupled multi-scale, multi-physics simulation framework for aluminium electrolysis. Appl. Math. Model..

[90] Vogel YB (2020). The corona of a surface bubble promotes electrochemical reactions. Nat. Commun.

[91] Son J (2012). A practical superhydrophilic self cleaning and antireflective surface for outdoor photovoltaic applications. Sol. Energy Mater. Sol. Cells.

[92] Anand U (2021). Dynamics of thin precursor film in wetting of nanopatterned surfaces. Proc. Natl Acad. Sci..

[93] Otitoju TA, Ahmad AL, Ooi BS (2017). Superhydrophilic (superwetting) surfaces: A review on fabrication and application. J. Ind. Eng. Chem..

[94] Ashkarran AA, Mohammadizadeh MR (2008). Superhydrophilicity of TiO_2_ thin films using TiCl_4_ as a precursor. Mater. Res. Bull..

[95] Kenanakis G (2008). Light-induced reversible hydrophilicity of ZnO structures grown by aqueous chemical growth. Appl. Surf. Sci..

[96] Feng X (2004). Reversible super-hydrophobicity to super-hydrophilicity transition of aligned ZnO nanorod films. J. Am. Chem. Soc..

[97] Hilner E (2009). Ordering of the nanoscale step morphology as a mechanism for droplet self-propulsion. Nano Lett..

[98] Launay G (2020). Self-propelled droplet transport on shaped-liquid surfaces. Sci. Rep..

[99] Antony, A. P. Design and fabrication of a water electrolysis unit for an integrated life support system (No. NASA-CR-66654). (Allis-Chalmers, Milwaukee, 1968).

[100] Jensen, F. C. & Schubert, F. H. Technology advancement of the static feed water electrolysis process (No. NASA-CR-151934). (Life Systems INC., Cleveland, 1977).

[101] Bastian JM (2021). Understanding the activity transport nexus in water and CO_2_ electrolysis: state of the art, challenges and perspectives. Chem. Eng. J..

[102] Narcy M, Colin C (2015). Two-phase pipe flow in microgravity with and without phase change: recent progress and future prospects. Interfacial Phenom..

[103] Tørklep K, Øye HA (1982). Viscostiy of molten alkaline-earth chlorides. J. Chem. Eng. Data.

[104] Ioroi T, Kitazawa N, Yasuda K, Yamamoto Y, Takenaka H (2001). IrO_2_-deposited Pt electrocatalysts for unitized regenerative polymer electrolyte fuel cells. J. Appl. Electrochem..

[105] Pettersson J, Ramsey B, Harrison D (2006). A review of the latest developments in electrodes for unitised regenerative polymer electrolyte fuel cells. J. Power Sources.

[106] Wu X, Scott K, Xie F, Alford N (2014). A reversible water electrolyser with porous PTFE based OH^-^ conductive membrane as energy storage cells. J. Power Sources.

[107] Landman A (2017). Photoelectrochemical water splitting in separate oxygen and hydrogen cells. Nat. Mater..

[108] Symes MD, Cronin L (2013). Decoupling hydrogen and oxygen evolution during electrolytic water splitting using an electron-coupled-proton buffer. Nat. Chem..

[109] Rausch B, Symes MD, Chisholm G, Cronin L (2014). Decoupled catalytic hydrogen evolution from a molecular metal oxide redox mediator in water splitting. Science.

[110] Bienvenu, G. Method for the Co-generation of electrical and hydrogen power. EP2460219 (A1), patent issued January 2^nd^, 2019. https://patents.google.com/patent/EP2460219A1/en (accessed 25 Nov 2022).

[111] Dotan H (2019). Decoupled hydrogen and oxygen evolution by a two-step electrochemical-chemical cycle for efficient overall water splitting. Nat. Energy.

[112] Hodges A (2022). A high-performance capillary-fed electrolysis cell promises more cost-competitive renewable hydrogen. Nat. Commun..

[113] Stiber S (2021). Porous transport layers for proton exchange membrane electrolysis under extreme conditions of current density, temperature, and pressure. Adv. Energy Mater..

[114] Kelsall G, Tang S, Yurdakul S, Smith AL (1996). Electrophoretic behaviour of bubbles in aqueous electrolytes. J. Chem. Soc. Faraday Trans..

